# Improving Public Health System Performance Through Multiorganizational Partnerships

**Published:** 2010-10-15

**Authors:** Glen P. Mays, F. Douglas Scutchfield

**Affiliations:** Department of Health Policy and Management, Fay W. Boozman College of Public Health, University of Arkansas for Medical Sciences; University of Kentucky, Lexington, Kentucky

## Abstract

Public health activities in the United States are delivered through multiple public and private organizations that vary widely in their resources, missions, and operations. Without strong coordination mechanisms, these delivery arrangements may perpetuate large gaps, inequities, and inefficiencies in public health activities. We examined evidence and uncertainties concerning the use of partnerships to improve the performance of the public health system, with a special focus on partnerships between public health agencies and health care organizations. We found that the types of partnerships likely to have the largest and most direct effects on population health are among the most difficult, and therefore least prevalent, forms of collaboration. High opportunity costs and weak and diffuse participation incentives hinder partnerships that focus on expanding effective prevention programs and policies. Targeted policy actions and leadership strategies are required to illuminate and enhance partnership incentives.

## Introduction

Public health activities in the United States are implemented through the combined actions of multiple government and private organizations that vary widely in missions, resources, and operations. Public health agencies serve as focal points, but these agencies rely heavily on their ability to inform and influence the work of others. Public health delivery systems thus are complex and adaptive systems that operate through the interactions of multiple heterogeneous actors. Without strong coordination mechanisms, these systems may perpetuate large gaps and inequities in the availability and effectiveness of public health activities and substantial inefficiencies in performance (1). In other sectors, interorganizational partnerships and alliances have been used to coordinate action in ways that improve information flow, reduce duplication of effort, achieve economies of scale and scope, and accelerate adoption of effective practices (2).

Recognizing these issues, the Institute of Medicine's 2003 review of the nation's public health system called for "a new generation of intersectoral partnerships" that span the many different sectors of organizational activity that affect population health and that coordinate activities across these sectors (3). Partnerships that integrate medical care and public health approaches to achieve comprehensive health improvement are particularly important. In this article, we examine evidence, uncertainties, and emerging opportunities regarding the use of partnerships to improve the public health system.

## Conceptual Framework: Partnerships as Collective Action

Public health partnerships are forms of collective action undertaken to promote health and prevent disease and injury in populations at risk. Collective action occurs when organizations agree to coordinate activities in pursuit of shared objectives (4). Partnerships may benefit member organizations by allowing them to share information and expertise, human and material resources, or intangibles such as reputation, trust, and visibility. Partnerships may allow organizations to combine operations and realize economies of scope and scale in the production of public health services. Similarly, partnerships may allow coordinated delivery of related programs and services, potentially resulting in a larger combined impact on population health. In these ways, partnerships allow organizations to pursue objectives that may not be possible through independent actions.

Partnership formation in public health depends on the range of organizations available in a given community and the ability and willingness of each organization to contribute to public health activities (5,6). For some activities, economic incentives may encourage organizations to contribute voluntarily — such as the opportunity to gain revenue, reduce costs, or achieve visibility and recognition that confers a political or marketing advantage (7,8). Many organizations also may have noneconomic motives to contribute, such as an altruistic mission to improve health and social welfare (9). Policy and regulatory actions, such as the requirement that tax-exempt hospitals meet community benefit standards, may motivate contributions. Like other public goods, however, public health activities may not generate sufficiently powerful incentives to ensure that they will be fully provided by voluntary action (10,11). In some cases, noncontributing organizations benefit from the public health activities performed by others, such as when health insurers realize cost savings from tobacco use cessation programs or vaccination programs (12). A traditional role for public health agencies is to directly provide beneficial activities that are underperformed by others, while also stimulating contributions by other organizations to minimize unfair benefits (5). An agency's success in these endeavors will influence partnership formation.

Concepts from behavioral economics suggest that collective actions may falter even when participation incentives are strong. Organizations often fail to value accurately the expected gains from collective action because of common decision errors, including inconsistent information, risk aversion, mistrust, and tendencies to favor the status quo (11). A fundamental challenge for public health professionals is to improve understanding of the expected value of partnerships among key stakeholders and to use policy and leadership strategies to enhance the incentives and blunt the disincentives for participation.

## Current Evidence and Uncertainties About Partnerships

### Partnership incentives

Partnerships provide a structure in which organizations can cooperate in producing activities designed to promote health and prevent disease and injury, but organizations will participate only if they have sufficient incentives. The perception of health care providers or payers that participation in a partnership will enhance revenues or reduce costs by increasing the reach and uptake of cost-effective prevention programs and services is an economic incentive. However, the magnitude, distribution, and timing of such financial gains or cost savings are areas of considerable uncertainty and depend heavily on the nature and success of the partnership (13,14). Partnerships designed to increase the reach of underused but highly cost-effective clinical preventive services — such as smoking cessation, influenza vaccination, aspirin use, colorectal cancer screening, or family planning services — may reduce future medical care costs, especially if the partnerships target services to the populations at risk and allow implementation costs to be shared among multiple organizations (15,16). Similarly, partnerships designed to increase implementation of and compliance with nonclinical public health programs and policies — such as smoking bans, seat belt laws, and environmental changes that promote nutrition and physical activity — may produce cost savings by reducing disease burden and the future need for medical care (17,18). Such partnerships for nonclinical interventions may have the added economic advantage of low implementation costs.

The strength of economic incentives for partnership formation depends not only on the magnitude of expected cost savings but also on the timing and distribution of these savings. Partnerships to promote colorectal screening, for example, involve time lags of a decade or more before cost savings from disease prevention can be expected, while partnerships that enhance tobacco control or vaccination coverage may generate a mix of short-term and longer-term savings. Time lags weaken the economic incentives for public health partnerships, especially for investor-owned organizations that operate under short-term financial expectations and for employers and health insurers that experience turnover in their covered populations over time (19). Health care payers such as health insurers, employers, Medicare, and Medicaid stand to gain most directly from partnerships that enhance the delivery of cost-effective preventive services under current payment policies. Some physicians and hospitals may lose revenue as a result of public health partnerships that reduce medical care use (20). On the other hand, some providers may realize savings from partnerships that target segments of the population that are uninsured and would otherwise require uncompensated medical care. The expected distribution of these economic gains and losses in a community shape economic motivations for participating in partnerships.

Research suggests that partnership incentives may depend partly on the size and market position of contributing organizations. Organizations that serve large segments of the community have strong incentives for partnership because they stand to gain large shares of any public goods produced through collective action (8,21). Small organizations may achieve economies of scale through partnerships by producing public health activities collaboratively that would be inefficient or unfeasible to produce independently (22). Organizations that fall between these 2 extremes may face diminished incentives.

Many organizations pursue public health partnerships primarily for noneconomic reasons, such as the desire to reach new target populations, expand the quantity or quality of services, and influence high-priority health issues. Noneconomic incentives often attract organizations with closely compatible missions, resulting in a preponderance of government and nonprofit participants in many public health partnerships (5,8). Partnerships that include both economic and noneconomic incentives may appeal to other participants.

### Partnership functions

Partnerships provide a structure for accomplishing several public health functions, including information exchange, planning and policy development, and implementation of programs and policies. Partnerships focus on information exchange by supporting surveillance, epidemiologic investigation, needs assessment, and research translation activities. Contemporary examples include sentinel provider networks for influenza, syndromic surveillance systems, and health registries such as those for monitoring cancer, vaccination, and communicable diseases. More recently, some communities have formed partnerships to support the exchange of electronic health information for clinical decision making as well as public health surveillance and research. Research suggests that the quality of information generated through such partnerships depends partly on the nature of the relationships among participants (23).

Planning and policy development partnerships promote coordination and reduce duplication among organizations that otherwise work independently. Often these partnerships form as a result of communitywide assessment and performance measurement processes that identify unmet needs and opportunities for coordination, such as the National Association of County and City Health Officials' Mobilizing for Action Through Planning and Partnerships program, or the Centers for Disease Control and Prevention's National Public Health Performance Standards program. In some cases, these partnerships also function as advocacy coalitions that develop and promote policy proposals of common interest (24). Tobacco control coalitions are successful contemporary examples that work to secure smoking restrictions and tobacco tax increases in many states and communities.

Implementation partnerships bring organizations together to collaborate in delivering public health interventions. The focus on implementation can allow these partnerships to have more direct and immediate health effects than those focused exclusively on information exchange and planning. However, the success of these endeavors hinges on their ability to focus on evidence-based interventions, target interventions tightly to populations at risk, and pursue implementation on a sufficiently large scale (17,18,25,26). Success is likely to depend heavily on information exchange and planning and policy development activities. For this reason, large-scale implementation partnerships often develop only after other, prerequisite forms of collaboration have succeeded (5). Additionally, these partnerships may demand more human and financial resources and require more sacrifice of organizational autonomy and control than other forms of collaboration. Consequently, participating organizations may face substantial opportunity costs — alternative pursuits and individual interests that must be sacrificed — to make these partnerships successful.

Some of the most successful implementation partnerships use external funding to diminish opportunity costs. Prominent examples include federally funded initiatives such as Steps to a HealthierUS, Racial and Ethnic Approaches to Community Health Across the U.S., and most recently Communities Putting Prevention to Work — all of which focus on preventing chronic diseases and reducing health disparities through community-level, multiorganizational actions. The realities of high operating costs but limited external funding mean that these types of partnerships reach a small number of communities nationwide. Moreover, the time-limited nature of external funding creates uncertainties about long-term sustainability of the partnership. Success in securing ongoing financial support and in expanding geographic reach depends heavily on the partnership's entrepreneurship and ability to document health and economic gains (13).

### Partnership composition and structure

Partnerships are social networks formed among organizations; consequently, the substantial body of knowledge about social network structure helps to elucidate these collaborations (27,28). Network breadth reflects the array of different actors, which determines the amount and type of organizational resources that may be contributed. Network density measures the amount of interconnectedness between organizations, which facilitates their ability to work together. Network centrality reflects the relative influence of a single organization within a partnership, which can be important for coordinating and focusing collaborative actions. Both theory and research suggest that these constructs may influence partnership functioning, but their magnitudes and mechanisms of effect in public health are largely unknown.

Evidence suggests that both the breadth of organizations contributing to public health activities and the scope of their participation has been increasing in recent years. A study of partnerships in US communities with at least 100,000 residents found significant increases in the types of organizations that participate in public health activities from 1998 to 2006 (29,30). Not surprisingly, local and state government agencies were among the most frequent contributors to public health partnerships (Table), but hospitals, physicians, community health centers, and universities significantly increased their participation over time.

Research also shows that public health partnerships generally adhere to 1 of 7 distinct structural configurations based on network breadth, density, and centrality (Figure) (29,30). Three of these configurations support a broad and comprehensive scope of public health activities, of which 1 configuration relies heavily on the work of government public health agencies and 2 others delegate considerable responsibility to other partner organizations. Two partnership configurations deliver an intermediate (conventional) scope of public health activities and differ primarily in the centrality of the local public health agency in these activities. The final 2 configurations deliver a limited scope of public health activities and differ in both the centrality and density. Partnerships frequently migrate from 1 configuration to another over time, with a trend toward supporting a broader scope of activities and engaging a wider range of organizations.

**Figure 1:**
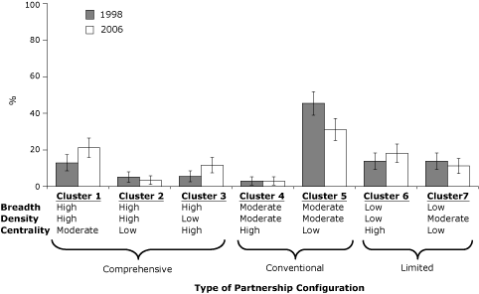
Prevalence of 7 public health partnership configurations, 1998 and 2006. Error bars represent 95% confidence intervals. Data were obtained from a survey of the 351 agencies that responded in both years (29,30). Seven configurations were identified through multivariate cluster analysis, each one distinguished by network breadth, density, and centrality. Breadth represents the array of actors involved in the partnerships; density represents the amount of interconnectedness between organizations; and centrality represents the relative influence of a single organization within a partnership.

**Table:** 

**Type of Partnership Configuration (Breadth/Density/Centrality)**	**Prevalence, % (95% CI)**	

	**1998**	**2006**
**Comprehensive**		
Cluster 1 (High/High/Moderate)	13 (9-17)	21 (16-27)
Cluster 2 (High/High/Low)	5 (2-8)	3 (1-6)
Cluster 3 (High/Low/High)	6 (3-9)	12 (8-16)
**Conventional**		
Cluster 4 (Moderate/Moderate/High)	3 (1-5)	3 (1-5)
Cluster 5 (Moderate/Moderate/Low)	45 (39-52)	31 (25-37)
**Limited**		
Cluster 6 (Low/Low/High)	14 (9-18)	18 (13-23)
Cluster 7 (Low/Moderate/Low)	14 (9-18)	11 (7-15)

Recent evidence suggests that partnerships operate somewhat differently in small and rural communities, where human and material resources are generally more limited. A recent network analysis of rural public health systems finds that smaller communities have fewer organizations available to address local health needs and therefore rely more heavily on the local public health agency to play central roles (31). In larger rural communities, public health partnerships tend to fragment into specialized collaborations, and the public health agency plays more peripheral roles. In the smallest communities, partnerships achieve more density when the local public health agency operates under centralized state governance, but in larger communities decentralized governance appears to foster denser partnerships, perhaps through enhanced autonomy and opportunities for entrepreneurship. These findings imply that partnership strategies should be tailored to the size of the community, the governance and legal environment for public health, and the types of activities to be undertaken through collective action. Considerable uncertainties remain about which partnership network structures work best in which public health settings.

### Partnership outcomes and impact

Evidence for the influence of public health partnerships on population health is limited but has grown in recent years alongside the larger evidence base supporting population-based disease prevention interventions (25). Measuring the effects of partnerships is complicated by the long time periods often required to change health behaviors and outcomes at a population level, the many confounding factors that simultaneously influence health endpoints of interest, and the fact that partnerships may have diffuse effects on multiple public health programs and outcomes. Nevertheless, a comprehensive evidence review found that among 34 reviewed studies of public health partnerships, 10 produced evidence of improved population health outcomes potentially attributable to partnerships, including such outcomes as incidence of lead poisoning, adolescent pregnancy, infant mortality, and motor vehicle crashes (32). Another 14 studies found evidence of behavior change attributable to partnership activity in areas such as tobacco use, alcohol use, physical activity, and safe sexual practices. The strongest of these studies, however, suggested that the effects on health behaviors may not be as large as intended (33). Another set of 22 studies suggested that partnerships generated beneficial changes in policies, programs, or environmental conditions such as the adoption of smoking bans, changes in school lunch menus, or the creation of exercise trails and community exercise groups (32). These types of partnership effects could be expected to produce population health improvements over time if appropriately sustained. However, these studies relied on case study research designs that could not establish definitively that observed changes were attributable to the partnerships. Nevertheless, this review and more recent studies collectively suggest that partnerships can produce beneficial outcomes under the right circumstances (34-36).

Evidence concerning the economic impact and cost-effectiveness of public health partnerships is an area largely unaddressed in the empiric literature, as is the more general question of the cost-effectiveness of community preventive services (13,14). Producing this evidence requires measuring the direct and indirect costs of participating in public health partnerships, including the opportunity costs that organizations incur. Obtaining valid measures of such costs is likely to require the active engagement of partnering organizations such as through practice-based research networks and participatory research methods. Such evidence is likely to be highly influential in shaping both government and private-sector decisions about contributing to partnerships.

## Policy Implications and Future Prospects

A growing body of evidence and experience suggests that multiorganizational partnerships are promising mechanisms for improving public health practice. However, the types of partnerships likely to have the most direct effects on population health are among the most difficult, and therefore least prevalent, forms of collaboration. These implementation partnerships are those that focus on expanding the reach of proven but underused interventions and policies through collaboration among public health agencies, health care organizations, and other stakeholders. To succeed in improving population health, such partnerships must target programs and policies tightly to populations at risk, implement activities on a sufficiently large scale, and maintain fidelity to key program and policy components over time. If successful, these partnerships can serve as vehicles for transforming public health practice from a diverse collection of activities and organizations into an organized and accountable delivery system for public health interventions.

Because the opportunity costs associated with these types of partnerships are high, policy and administrative actions are needed to strengthen the incentives for partnership formation. Better systems for measuring and reporting on the delivery of effective prevention programs and policies at the community level are needed to raise awareness of gaps in implementation and opportunities for collaboration. Accreditation systems and performance standards that are being developed for government public health agencies can be tailored to create incentives for partnerships (37). Moreover, the 2010 federal health reform law creates opportunities for adapting both medical care and public health funding streams to reward partnerships that expand the implementation of effective but underused prevention strategies. Collectively, these changes could serve as incremental steps along a path toward the more comprehensive pay-for-population health approaches that realign incentives for health improvement (38).

Beyond incentives, successful partnerships are likely to require changes in organizational culture, values, and strategy that can be achieved only through strong organizational leadership. Partnerships require leaders who can elucidate the participation incentives and constraints faced by individual organizations and identify shared objectives and compatible interests. Collaborative leadership can reveal the potential gains from partnerships and help organizations commit to difficult but beneficial public health actions that cannot be accomplished through independent endeavors.

## Acknowledgments

This paper was originally prepared for the University of Wisconsin's Mobilizing Action Toward Community Health (MATCH) project funded by the Robert Wood Johnson Foundation. Support for this research was provided by the Robert Wood Johnson Foundation's Public Health Practice-Based Research Networks Program (award no. 64676). Dr Mays also was supported through a Clinical and Translational Science Award from the National Center for Research Resources (award no. 1UL1RR029884).

## Figures and Tables

**Table.** Partnerships Between Local Public Health Agencies and Selected Organizations, 1998 and 2006 a

Type of Organization	**Agencies Reporting Partnerships^b^ With Selected Organizations,**N = 351			**Scope of Activity^c^ in Partnerships**		

	**1998,** No. (%)	**2006,** No. (%)	***P* Value^d^**	**1998, %**	**2006, %**	***P* Value^e^**
State government agencies	343 (98)	348 (99)	.20	37	47	.01
Local government agencies	322 (92)	339 (97)	.02	32	51	.001
Federal government agencies	155 (44)	215 (61)	.001	7	12	.04
Physician organizations	299 (85)	325 (93)	.006	20	24	.27
Hospitals	339 (97)	351 (100)	.004	37	41	.40
Community health centers	179 (51)	297 (85)	.001	12	29	.001
Nonprofit organizations	334 (95)	335 (95)	.95	32	34	.60
Faith-based organizations	NA^f^	286 (82)	NC	NA^f^	19	NC
Community-based organizations	NA^f^	325 (93)	NC	NA^f^	32	NC
Health insurers	159 (45)	186 (53)	.07	9	10	.57
Universities	230 (66)	275 (78)	.001	16	22	.07
Schools	NA^f^	315 (90)	NC	NA^f^	28	NC
Employers and business groups	NA^f^	269 (77)	NC	NA^f^	17	NC

Abbreviations: NA, not assessed; NC, not calculated.

a Data were obtained from a survey of all US local public health agencies that serve communities with at least 100,000 residents (29,30). These 497 agencies represent approximately 17% of all local public health agencies nationally but serve approximately 70% of the US population. Each agency was surveyed in the fall of 1998 (78% response rate) and again in the fall of 2006 (70% response rate). Data pertain to the 351 agencies that responded in both years.

b Defined as participating in 1 or more of 20 core public health activities.

c Defined as the mean proportion of activities undertaken through partnerships, based on a list of 20 core public health activities.

d Calculated by using χ^2^ test.

e Calculated by using equality of proportions test.

f Data element was collected in 2006 only.
